# Enhanced supercapacitor materials from pyrolyzed algae and graphene composites

**DOI:** 10.1038/s41598-023-48166-6

**Published:** 2023-12-01

**Authors:** Mariusz Szkoda, Malgorzata Skorupska, Jerzy P. Łukaszewicz, Anna Ilnicka

**Affiliations:** 1 Faculty of Chemistry, Department of Chemistry and Technology of Functional Materials, Gdańsk University of Technology, Narutowicza 11/12, 80-233 Gdańsk, Poland; 2 Advanced Materials Center, Gdańsk University of Technology, Narutowicza 11/12, 80-233 Gdańsk, Poland; 3 Faculty of Chemistry, Nicolaus Copernicus University in Torun, Gagarina 7, 87-100, Torun, Poland; 4 Centre for Modern Interdisciplinary Technologies, Nicolaus Copernicus University in Torun, Wilenska 4, 87-100 Torun, Poland

**Keywords:** Materials for energy and catalysis, Electrochemistry, Energy

## Abstract

This study focuses on the synthesis and characterization of supercapacitor materials derived from pyrolyzed natural compounds. Four compounds were investigated: methylcellulose with lysine (ML), methylcellulose with lysine-graphene composite (MLG), algae (A), and algae-graphene composite (AG). The pyrolysis process was utilized to convert these natural compounds into carbon-based materials suitable for supercapacitor applications. The properties of the resulting materials were analyzed extensively to evaluate their potential as supercapacitor electrodes. The electrochemical performance, including specific capacitance, cyclic stability, and rate capability was measured using various characterization techniques. The effects of incorporating graphene into the lysine-methylcellulose and algae matrices were also studied to explore the enhancements in supercapacitor performance. In both cases, the addition of graphene resulted in a positive effect. Among all the materials investigated, the algae-graphene composite exhibited the most favorable properties, demonstrating a specific capacitance of 192 F g^−1^ after 10,000 galvanostatic charge–discharge cycles at a current of 5 A g^−1^ in K_2_SO_4_ electrolyte. This exceptional performance underscores the potential of the algae-graphene composite as a highly efficient and durable electrode material for supercapacitor applications.

## Introduction

Supercapacitors, also known as electrochemical capacitors or ultracapacitors, have gained significant attention as promising energy storage devices due to their high power density, fast charge–discharge rates, and long cycle life compared to traditional batteries^1–4^. They have the potential to bridge the gap between conventional capacitors and batteries, offering a unique combination of energy and power delivery for various applications, including portable electronics, electric vehicles, and renewable energy systems^1,5^. The performance of supercapacitors relies heavily on the properties of the electrode materials^2^. Carbon-based materials have been widely explored as electrode materials for supercapacitors due to their high surface area, good electrical conductivity, and excellent electrochemical stability^6–10^. Traditional carbon sources, such as activated carbon, have been extensively utilized^11,12^. However, there is growing interest in developing sustainable and cost-effective electrode materials derived from natural compounds^13–16^. Pyrolysis, a thermal decomposition process in the absence of oxygen, has emerged as a promising method for synthesizing carbon-based materials from natural precursors. Pyrolyzed materials exhibit unique structural and electrochemical properties, making them attractive options for supercapacitor applications^17,18^. The use of natural compounds as precursors offers several advantages, including abundant availability, low cost, and environmental friendliness^19^.

Our study is situated within the context of the latest advancements in pyrolyzing diverse natural sources to serve as supercapacitor electrodes. Recent research has witnessed significant strides in this domain, showcasing the remarkable potential of materials derived from natural compounds. For instance, studies by Liu et al.^20^ demonstrated that pyrolyzed rice husks exhibited a specific capacitance of 278 F g^−1^ in a potassium hydroxide electrolyte, highlighting the impressive electrochemical performance achievable with bio-waste materials. Furthermore, Lv and colleagues^21^ explored the pyrolysis of chitosan, resulting in carbon-based materials with a specific capacitance of 306.4 F g^−1^ in 6 M KOH electrolyte, thus emphasizing the versatility of natural polymers for supercapacitor applications. Additionally, the work of Yi et al.^22^ on the pyrolysis of lignin-based materials showed a specific capacitance of 133 F g^−1^, demonstrating the viability of lignocellulosic biomass as a renewable electrode precursor. These examples underscore the burgeoning interest in sustainable and environmentally friendly electrode materials derived from readily available natural sources. The continuous investigation into the electrochemical behavior and properties of these materials promises to propel energy storage technology to new heights, with potential applications spanning a wide range of industries.

In this study, the synthesis and characterization of supercapacitor materials derived from pyrolyzed natural compounds was conducted. Specifically, four compounds were investigated: lysine with methylcellulose, lysine with methylcellulose-graphene composite, algae, and algae-graphene composite. Lysine, an essential amino acid, and methylcellulose, a biodegradable polymer, have shown potential in various applications due to their unique physicochemical properties. Algae, on the other hand, are abundant and renewable biomass resources that have gained attention for their potential use in energy storage systems. The incorporation of graphene, a two-dimensional carbon material with exceptional electrical conductivity and mechanical strength, offers the opportunity to enhance the performance of the resulting supercapacitor materials. Additionally, comprehensive studies utilizing solid-state physics techniques will be conducted alongside the electrochemical investigations. These investigations will provide valuable insights into the structural and morphological properties of the pyrolyzed natural compounds and their composites. Techniques such as scanning electron microscopy (SEM), Raman spectroscopy, X-ray photoelectron spectroscopy (XPS), nitrogen adsorption at a low temperature, and elementary analysis will be employed to characterize the crystalline structure, surface morphology, and nanoscale features of the materials. These complementary studies will offer a comprehensive understanding of the relationship between the structural properties and the electrochemical performance of the synthesized materials, facilitating the design and optimization of advanced supercapacitor materials. This study aims to investigate the electrochemical properties of these pyrolyzed natural compounds and evaluate their potential as electrode materials for supercapacitors. By analyzing the specific capacitance and cyclic stability of the synthesized materials, we strive to gain insights into the performance enhancements achieved by incorporating graphene and utilizing different natural precursors.

## Results and discussion

### Materials characterization

**Figure 1:**
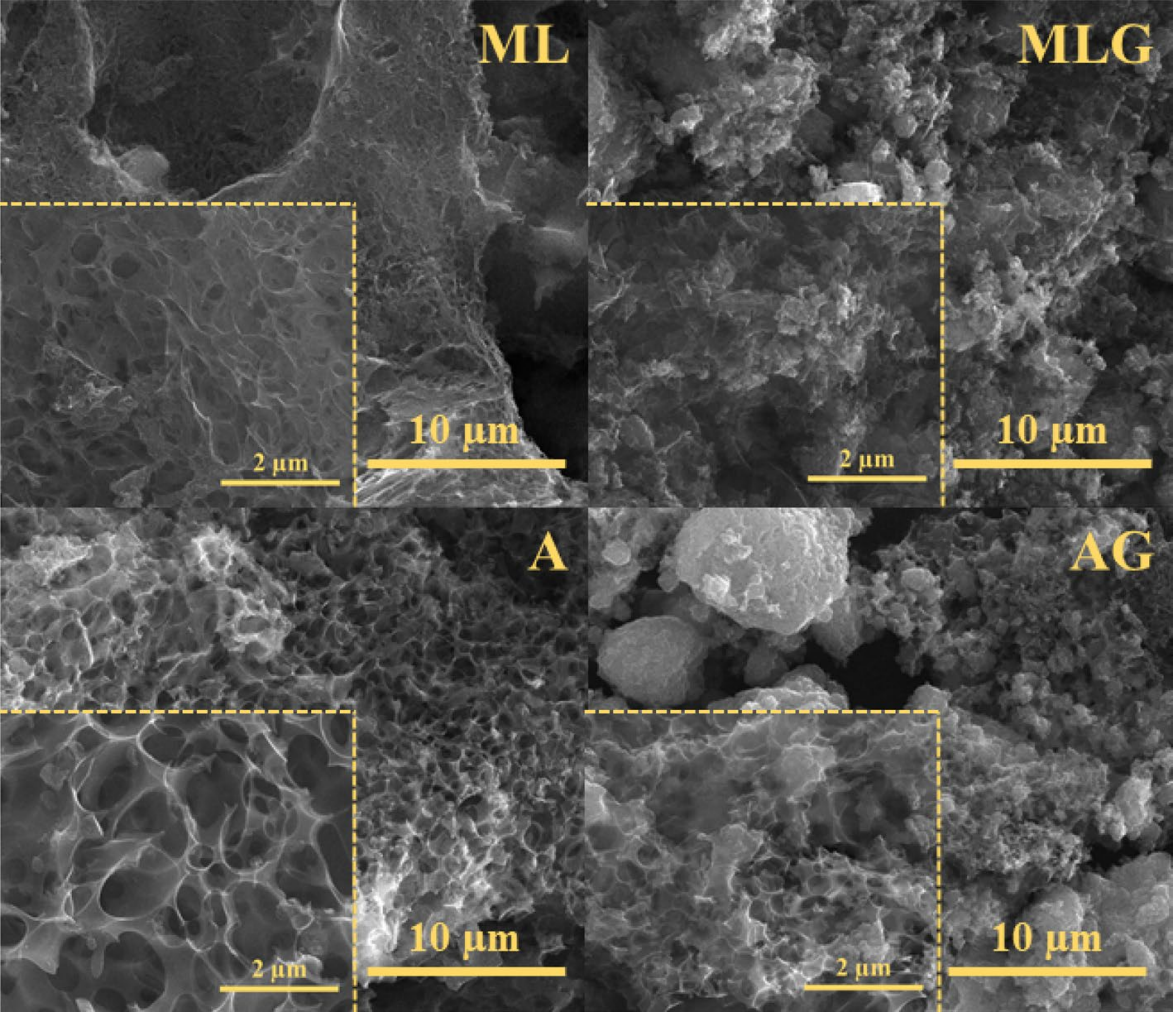
SEM images of ML, MLG, A, and AG samples with different magnifications.

SEM images provide crucial insights into the size, shape, and internal structure of materials. In this study, SEM analysis was utilized to characterize the morphology of the composite materials, and the results are presented in Fig. 1. Analyzing the SEM images, it becomes evident that the materials exhibit diverse surface morphologies, which can be attributed to the variations in the reactants used during the synthesis process. The composite materials obtained using different precursors demonstrate distinct structural changes, especially influenced by the presence of graphene. The SEM images reveal that the synthesis of the materials yielded irregularly shaped carbon structures with a porous surface for both methylcellulose (ML) and algae (A). Additionally, on the surface of these materials, small agglomerations of carbon particles corresponding to graphene are evident, clearly visible in the SEM images of methylcellulose-graphene (MLG) and algae-graphene (AG) composites. The presence of graphene in the composite materials is likely responsible for the observed changes in the morphology. The porous surface of the carbon materials is significant for supercapacitor applications, as it provides ample active sites for charge storage and efficient ion diffusion. Moreover, incorporating graphene adds to these materials' electrical conductivity, facilitating rapid electron transport during charge–discharge cycles.

**Figure 2:**
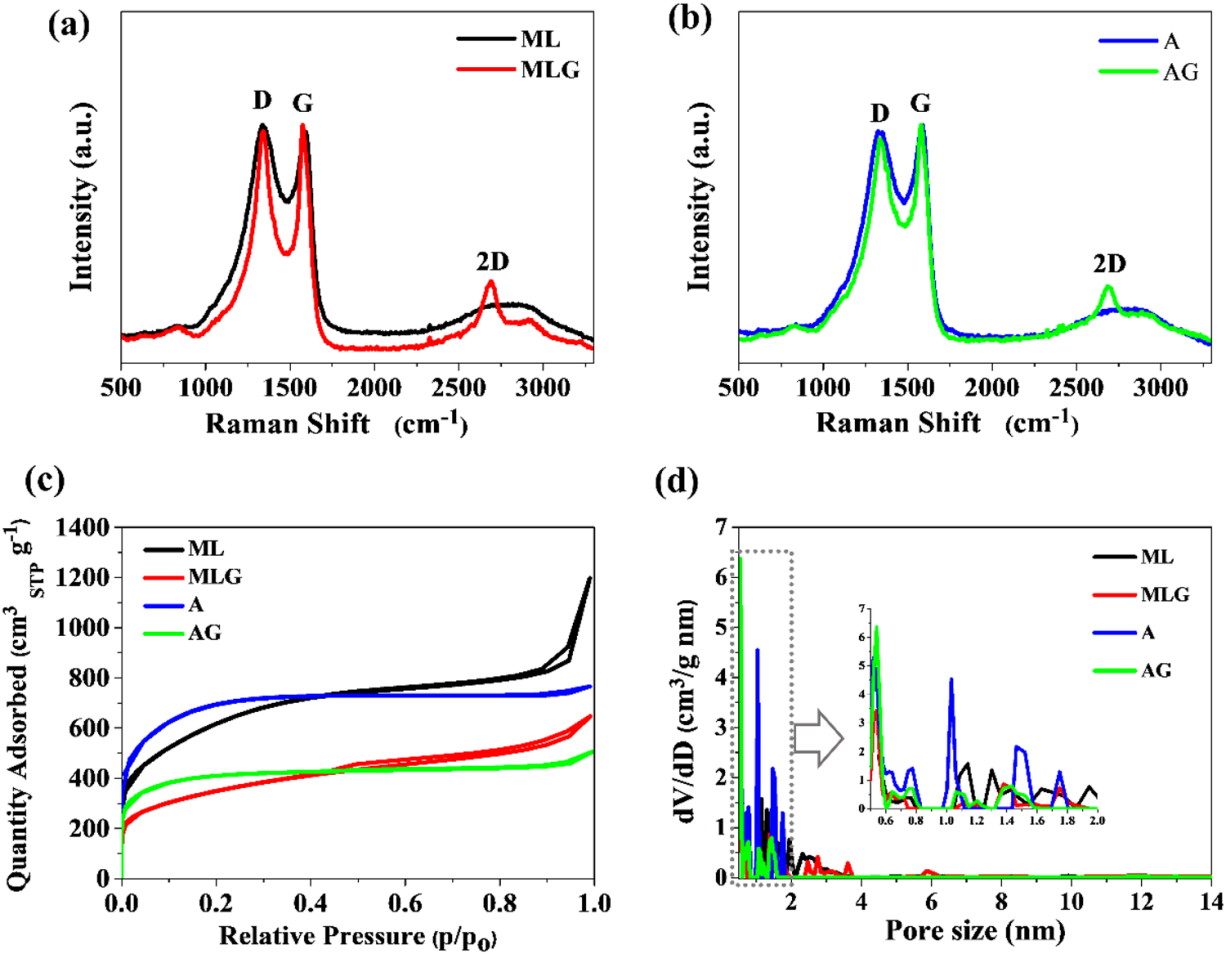
(**a**) and (**b**) Raman spectra, (**c**) N_2_ adsorption isotherms, (**d**) Pore size distributions calculated from N_2_ desorption curves for samples ML, MLG, A, and AG.

Raman spectroscopy is a leading method used to characterize carbon materials^23,24^. The disorder-induced band resulting from vacancies or dislocations in the graphene layer and at its edge (D-band), the band associated with graphene in graphite (G-band), and the band related to the graphene layer (2D-band) observed in the Raman spectra (Fig. 2a,b) were thoroughly analyzed. The intensities of these bands were determined at X-ray shift values of approximately 1335, 1583, and 2687 cm^−1^, respectively. The ratio of the D and G bands intensities allows determining the number of defects present in the structure of the analyzed carbon material. The I_D_/I_G_ ratio results for ML, MLG, A, and AG are 1.03, 0.98, 0.98, and 0.94, respectively. The D to G band intensity ratio reveals the structural changes and relative disorder-to-order ratio in the carbons obtained from activation and high-temperature heating. In the next stage, the number of graphene layers in the samples was determined by analyzing the ratio of 2D to G band intensities (I_2D_/I_G_). The ratio of 2D to G band intensities for ML, MLG, A, and AG is 0.31, 0.40, 0.30, and 0.36, respectively. This I_2D_/I_G_ intensity ratio is less than 1, indicating the presence of a few graphene layers.

The specific surface area of ML, MLG, A, and AG samples was determined using the Brunauer–Emmett–Teller (BET) method. The ML and A samples exhibited the highest specific surface areas when nitrogen-doped carbons obtained from methylcellulose with lysine or algae were used as substrates, with BET values of 2,049 m^2^ g^−1^ and 2,478 m^2^ g^−1^, respectively. The total pore volume determined at maximum p/p_o_ for the samples with the highest S_BET_ is 1.82 cm^3^ g^−1^ and 1.11 cm^3^ g^−1^ for samples ML and A, respectively. The physical morphology of the two samples synthesized with graphene was decreased to 1,184 m^2^ g^−1^ and 1,518 m^2^ g^−1^ for MLG and AG, respectively. However, this S_BET_ for MLG and AG is still very high, and the total pore volume was 0.99 cm^3^ g^−1^ and 0.75 cm^3^ g^−1^, respectively. The shape of the obtained nitrogen adsorption isotherms (Fig. 2c) measured at 77 K indicated the microporous structure of the obtained carbons (type I isotherms according to the IUPAC classification^25^) with a small hysteresis loop at a relative pressure of 0.4–1.0. The large surface area and significant share of micropore size (less than 2 nm) visible on pore size distribution curves (Fig. 2d) ensure full exposure of active sites, which is responsible for achieving high supercapacitor performance. Their structural properties will potentially facilitate the rapid transport of ions and electrons in the electrolyte throughout the carbon electrode matrix, which has a potentially high specific capacity. This aspect will be extensively explored in electrochemical measurements.

The elemental composition of samples ML, MLG, A, and AG, with or without the participation of commercial graphene, was determined using an elemental combustion analysis. Similar levels of carbon values were obtained for ML (78.10% by weight) and A (80.87% by weight) samples, but the highest carbon content was characterized by samples whose synthesis used graphene MLG (90.38% by weight) and AG (84.14% by weight). The addition of commercial graphene during the synthesis contributed to obtaining a material with a carbon content higher by 4 or 16% compared to the material where commercial graphene was introduced mechanically before the carbonization process. The nitrogen content of lysine and algae as good nitrogen-doped carbon precursors is very high after high-temperature treatment, at 4.71% by weight and 3.75% by weight for ML and A, respectively. On the other hand, the nitrogen content decreased for samples with graphene to 1.79% and 1.39% by weight for MLG and AG, respectively. For all analyzed samples, the hydrogen content reaches low values from 0.94 to 1.06% by weight. The percentages of elements obtained by elemental analysis are shown in Table 1.

**Table 1:** Elemental analysis of obtained carbon base materials (wt. %).

Sample	N (%)	C (%)	H (%)
ML	4.71	78.10	0.96
MLG	1.79	90.38	0.94
A	3.75	80.87	1.05
AG	1.39	84.14	1.06

**Figure 3:**
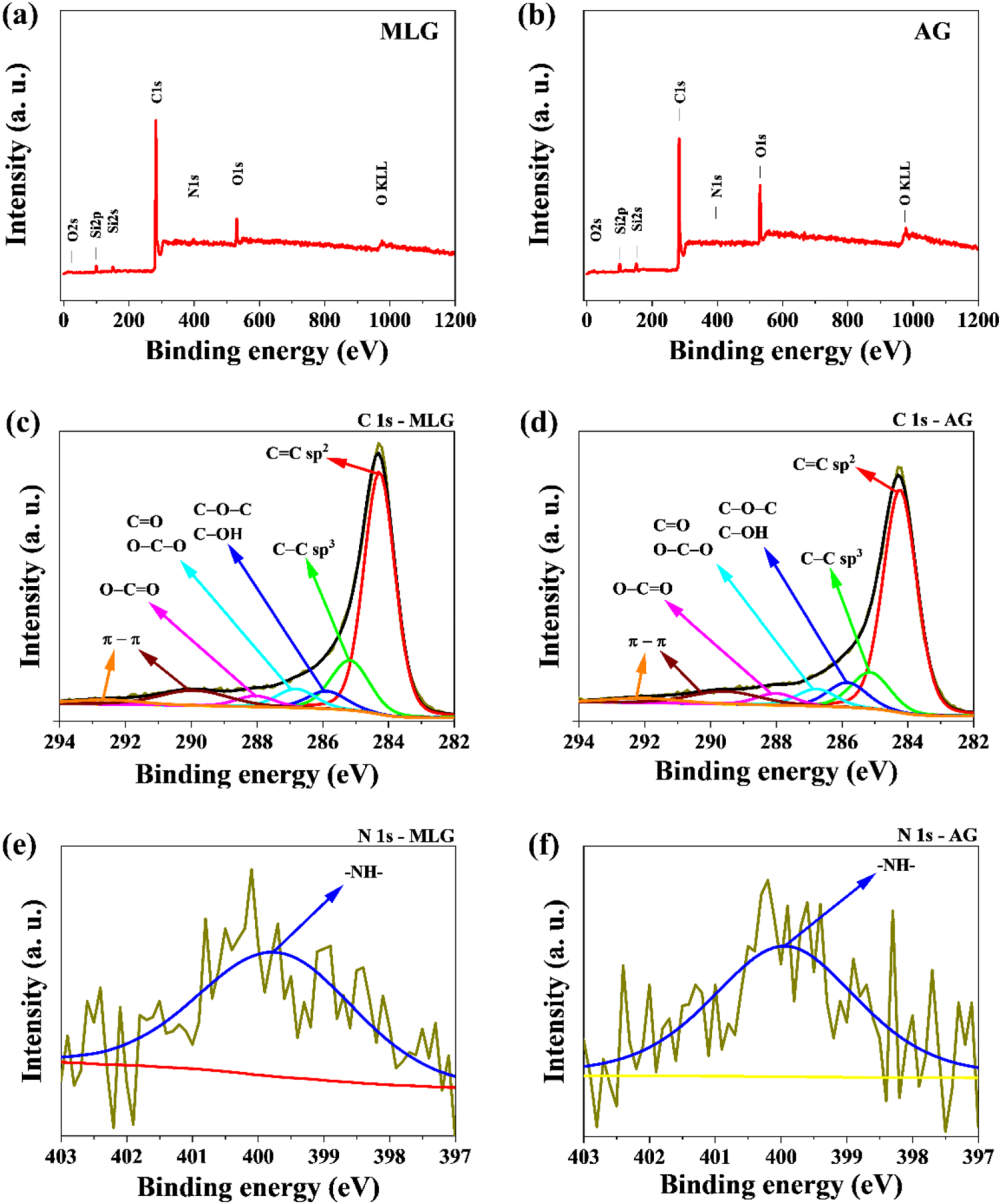
(**a**,**b**) Survey XPS spectra of MLG and AG hybrid materials; High-resolution XPS spectra of (**c**,**d**) C1s, (**e**,**f**) N1s orbitals.

To confirm the chemical state of the atoms present on the surface of the materials obtained, X-ray photoelectron spectroscopy analysis was performed. The low-resolution broad-band spectra for MLG and AG hybrid materials are shown in the energy range from 0 to 1,200 eV (Fig. 3a,b).

The deconvolution of the high-resolution spectra indicates the occurrence of elements in various chemical surroundings, which allows us to analyze the type of chemical bonds formed, characterized by a specific value of binding energy. The spectra of C1s, seen in Fig. 3c,d, made it possible to determine that C=C-type bonds (sp^2^) occurring at a bond energy of 284.3 eV are present in the studied structure. The energy band at 285.0 eV indicates the presence of a C–C bond (sp^3^). C–O–C and/or C–OH type bonds are also present in the obtained materials, which was confirmed by a band at a binding energy of 285.9 eV. The band at energies equal to 286.8 eV corresponds to C=O and/or O–C–O bonds, and at energy 288.0 eV is characteristic of O–C=O-type bonds. In addition, the band at energies equal to 289.7 eV and 292.1 eV can be attributed to shake-up excitation from sp^2^-type carbon. The XPS spectrum for the tested samples in the N1s region (Fig. 3e,f) was fitted with one line at a binding energy of 399.7 eV corresponding to the pyrrolic-N bond.

### Electrochemical performance

#### Three-electrode configuration

**Figure 4:**
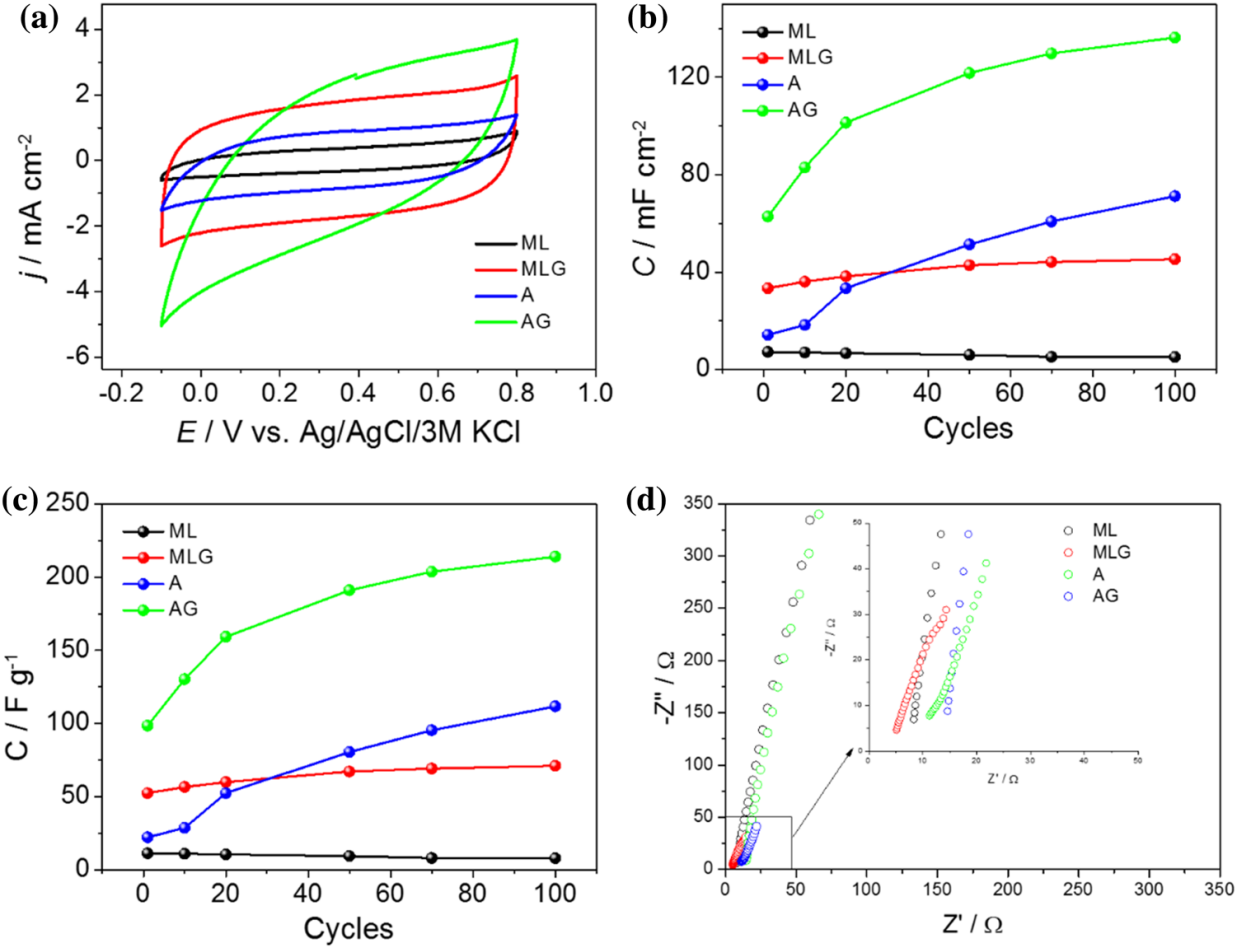
(**a**) Cyclic voltammetry curves recorded for N-doped carbon materials in 0.2 M K_2_SO_4_ with a potential window between − 0.1 V and + 0.8 V (v = 50 mV s^−1^). Specific capacitance plotted as a function of the number of cycles for investigated carbon materials: (**b**) per cm^2^ and (**c**) per gram. (**d**) Electrochemical impedance analysis of the obtained electrode materials in a three-electrode configuration at an open circuit potential (in a frequency range of 20 kHz to 1 Hz).

Various electrochemical measurements were conducted to evaluate the performance of porous carbons with and without graphene as electrode materials for supercapacitors, including cyclic voltammetry (CV) and galvanostatic charge–discharge (GDC) experiments. A three-electrode system was employed, comprising an Ag/AgCl/3 M KCl reference electrode, the obtained carbon-based materials as the working electrode, and a Pt mesh counter electrode. The electrochemical measurements were carried out using a 0.2 M K_2_SO_4_ neutral aqueous electrolyte. Cyclic voltammetry measurements were performed on four different electrode samples: methylcellulose with lysine (ML), methylcellulose with lysine-graphene composite (MLG), algae (A), and algae-graphene composite (AG). The CV curves for each sample were recorded within a voltage window of − 0.1 V to + 0.8 V (Fig. 4a). The obtained CV curves for all the samples exhibited an approximately rectangular shape, indicating the coexistence of electric double-layer capacitance (EDLC) and a reversible Faraday effect. This behavior is primarily attributed to the presence of heteroatoms or functional groups on the surface of the carbon materials^16,26–28^. The rectangular shape of the CV curves suggests efficient charge storage within the electric double layer, indicating good capacitive behavior of the electrodes^28–30^. Furthermore, the reversible Faraday effect observed in the CV curves indicates the occurrence of redox reactions associated with the presence of functional groups or heteroatoms on the carbon materials^31–33^. These redox reactions contribute to the overall charge storage capacity and enhance the electrochemical performance of the electrodes^34^. The rectangular CV curves and the presence of reversible Faraday effects suggest that the carbon materials, both with and without graphene, exhibit favorable electrochemical properties suitable for supercapacitor applications. The combination of electric double-layer capacitance and Faraday effects provides a synergistic effect, enabling efficient charge storage and high capacitance.

One intriguing finding is the significant enhancement in electrochemical capacitance observed for materials incorporating graphene despite having a smaller specific surface area than their counterparts without graphene. This unexpected observation suggests that the increase in specific capacitance is not solely reliant on the expansion of the surface area (S_BET_)^35,36^. Traditional supercapacitor research has sought to maximize surface area to achieve higher capacitance. However, the diminishing returns associated with this approach have become evident as the theoretical maximum limit for surface area utilization seems to have been reached^35^. The discovery that materials with graphene additives exhibit improved capacitance, even with a smaller surface area, challenges the notion that surface area is the sole driver of enhanced performance. Other factors, such as the conductivity and structural characteristics introduced by graphene, may contribute to the increased electrochemical capacitance^36^. The exceptional electrical conductivity of graphene facilitates efficient charge transfer and electron transport within the electrode, enabling a higher capacitive response. Additionally, the unique structural properties of graphene, such as its two-dimensional layered structure and large aspect ratio, may create favorable conditions for ion diffusion and accessibility to active sites, thereby enhancing the charge storage capability^37,38^. Therefore, the race for achieving the highest surface area is no longer the sole priority in supercapacitor research. It has become increasingly evident that, besides specific surface area, other factors play a crucial role in determining the capacitance of supercapacitor materials. These factors include the distribution of pore sizes within the material and, equally importantly, the nature of the electrolyte used. The latter consideration is particularly significant, as it involves matching the size of the ions in the electrolyte to the pore size within the material. In this context, optimizing the pore size distribution has gained prominence because it influences ion transport and accessibility to the electrode's surface. A well-balanced pore structure can facilitate efficient ion diffusion, leading to enhanced capacitance and improved charge–discharge performance. Furthermore, the choice of electrolyte plays a pivotal role in dictating the supercapacitor's overall performance. The ion size in the electrolyte should ideally align with the pore size distribution within the electrode material. This alignment ensures that ions can readily access and interact with the available surface area, maximizing the electrochemical capacitance. In essence, achieving high capacitance in supercapacitors is not solely about increasing the surface area; it also involves optimizing pore size distribution and carefully selecting an electrolyte that matches the pore size characteristics, creating an effective synergy between these factors for superior energy storage capabilities. Thus, the incorporation of graphene has opened up new avenues for improving electrochemical performance, demonstrating that factors beyond surface area can significantly influence the capacitance of electrode materials. Moreover, among the investigated materials, the highest capacitance after 100 charge–discharge cycles was observed for the material containing pyrolyzed algae with graphene (Fig. 4b,c).

Figure 4d displays the electrochemical impedance spectra recorded for the various carbon-based materials (ML, MLG, A, AG) using a three-electrode configuration at an open circuit potential, covering a frequency range from 20 kHz to 1 Hz. No semicircle was observed in the high-frequency region, which typically represents the charge transfer resistance at the electrode–electrolyte interface.

This absence of a semicircle indicates a relatively small charge transfer resistance, which can be attributed to the porous structure of the materials^39^. Moreover, in the low-frequency region, a noteworthy trend emerged. The decreased slope observed in this region suggests a transition from purely capacitive to pseudocapacitive behavior. This transition often indicates the presence of faradaic processes at the electrode surface, which contribute to the overall capacitance. It was noted that the addition of graphene (as seen in the MLG and AG materials) resulted in a reduction in resistance. This finding underscores the positive impact of graphene on enhancing charge transfer kinetics within the electrode–electrolyte interface, which is valuable for improving the overall performance of supercapacitors. This specific combination exhibited exceptional electrochemical performance, highlighting the synergistic effects between pyrolyzed algae and graphene in terms of charge storage. In each case, an increase in capacitance was observed with successive cycles, indicating a progressive improvement in the electrochemical performance of the materials. However, to gain a deeper understanding of this phenomenon, materials containing graphene were chosen for subsequent studies.

**Figure 5:**
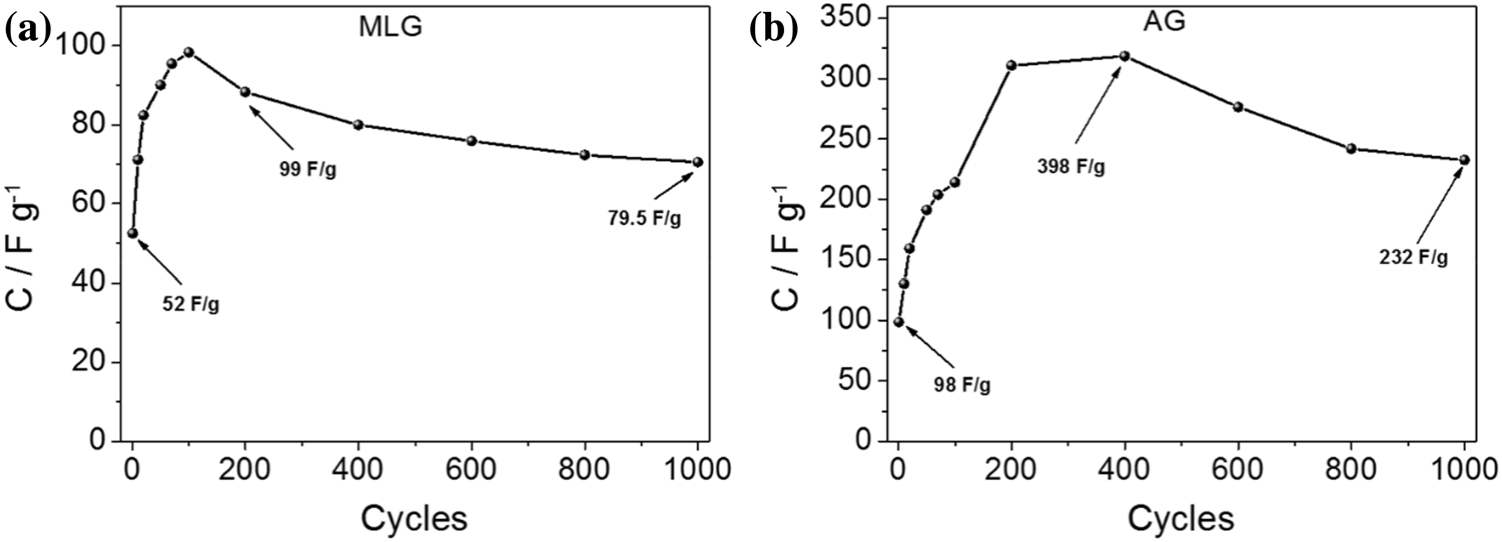
Curves of specific capacitance plotted as a function of cycle number for (**a**) MLG and (**b**) AG (1,000 cycles).

To assess the long-term repeatability of the observed phenomenon and to determine if the capacitance value continues to increase over an extended period, electrochemical measurements were conducted over 1,000 charge–discharge cycles. The results are depicted in Fig. 5. For the material with methylcellulose-graphene composite (MLG), an increase in capacitance value was observed during the initial 100 cycles. Similarly, for the material with algae-graphene composite (AG), an increase in capacitance was observed for the first 400 cycles. Subsequently, a gradual decrease in capacitance was observed for both MLG and AG. Furthermore, after 1,000 cycles, the capacitance level was still exceptional and higher than the initial value. This indicates the excellent cycling stability and long-term performance retention of the materials with graphene additives. These findings provide solid evidence that the observed increase in capacitance value over cycling is a replicable phenomenon. Moreover, they demonstrate the ability of the materials, even with the subsequent decrease in capacitance, to maintain outstanding performance over an extended period. The initial increase, followed by a gradual decrease in electrochemical capacitance during charge–discharge cycles for carbon-based materials may be attributed to several factors. During the initial cycles, the electrode materials undergo activation processes, such as forming an electrochemical double layer and rearranging surface functional groups. This leads to an increase in available active sites for charge storage and an enhancement in electrochemical performance, resulting in a higher capacitance. However, as the cycling progresses, certain phenomena can occur that contribute to a decrease in capacitance. One of the main factors is the potential for electrode degradation over prolonged cycling. Continuous charge–discharge cycles can lead to structural changes, such as the formation of cracks or the degradation of the carbon framework, which can impede charge transport and reduce the effective surface area accessible for electrochemical reactions. Additionally, the presence of irreversible Faradaic reactions can contribute to capacitance decay. Faradaic reactions involve charge transfer processes that result in the formation of new chemical species or the decomposition of the electrolyte, which can diminish the overall capacitance over time^38,40^.

#### Two-electrode configuration

**Figure 6:**
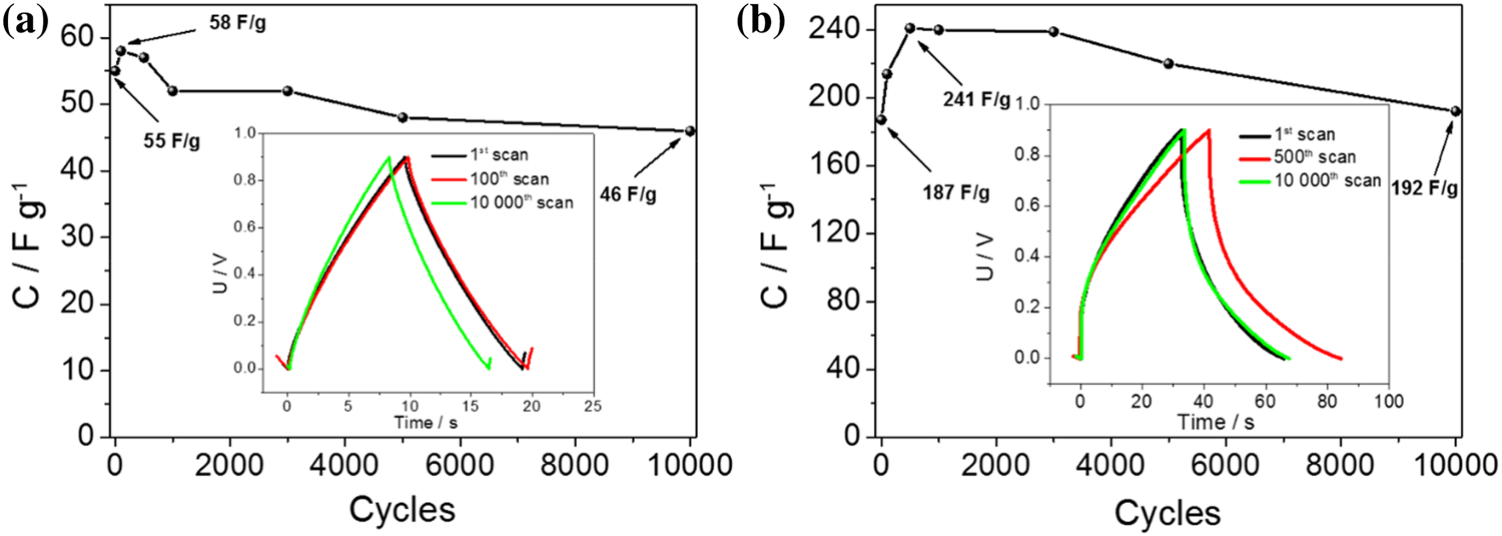
Curves of specific capacitance plotted as a function of cycle number for (**a**) MLG and (**b**) AG. Insets: exemplary galvanostatic charge–discharge curves for the electrode materials recorded at 5 A g^−1^.

To evaluate the stability of symmetric supercapacitors based on the materials with methylcellulose-graphene composite and algae-graphene composite, multiple charge–discharge cycles were conducted in a two-electrode configuration. The supercapacitors underwent an extensive cycling test of 10,000 cycles to assess their long-term stability and performance. Figure 6a,b present the galvanostatic charge–discharge (GCD) curves recorded at a current density of 5 A g^−1^ for MLG and AG, respectively. These curves provide insights into the charge storage and discharge behavior of the supercapacitor electrodes over a long cycling period. In the two-electrode configuration, it is observed that there is no significant increase in capacitance with successive cycles, as previously observed in the three-electrode setup. However, it is worth noting that there is still an increase in capacitance throughout the cycling test, especially up to approximately 500 cycles.

The absence of a pronounced capacitance increase in the two-electrode configuration can be attributed to several factors. Firstly, the closer proximity between the two electrodes in this setup may result in a limited diffusion of ions and a reduced accessibility of active sites, thus inhibiting the full utilization of the electrode materials' capacitance. Moreover, a vacuum sealer is commonly used during the fabrication of prototype supercapacitors, which likely leads to a quicker impregnation of the carbon electrodes with the electrolyte. This accelerated impregnation may result in a faster initial capacitance increase during the early cycles.

Despite the absence of a continuous capacitance increase, the observed retention of capacitance up to around 10,000 cycles indicates the stability and durability of the MLG and AG materials in the symmetric supercapacitor configuration. This capacitance retention suggests that the materials can still provide reliable and sustainable energy storage performance over several cycles.

Among the investigated materials, the AG (algae-graphene composite) material exhibited the highest capacitance, reaching a maximum value of 241 F g^−1^. The combination of algae and graphene in the composite material seems synergistic, leading to an enhanced capacitance compared to the other studied materials. The presence of graphene contributes to improved electrical conductivity, facilitating efficient charge transfer and higher charge storage capacity. Meanwhile, the unique properties of algae, such as its high surface area and porosity, likely provide additional active sites for electrochemical reactions, further enhancing the capacitance^41–43^. For comparative analysis, three representative charge–discharge cycles are presented: the first cycle, the cycle at which the maximum electrochemical capacity was achieved, and the final cycle. These cycles offer insights into the electrochemical behavior and performance of the studied materials over the course of the testing (see Fig. 6 inset). In Table 2, the electrochemical capacitances of the obtained best-performing material were compared with literature values for carbon materials derived from natural compounds.

**Table 2:** Activated carbon based on various precursors as supercapacitor electrodes in a two-electrode system.

Electrode material	C_s_ (F g^−1^)	Current density or scan rate	Refs.
Carbon from pistachio nutshell	261	0.2 A g^−1^	^44^
Carbon from pyrolysis of biomass wastes	223.9	5 mV s^−1^	^45^
Carbon from pyrolysis of seaweeds	203	2 mV s^−1^	^46^
Carbon from pyrolysis of waste polycarbonate	182.1	1 A g^−1^	^47^
Carbon from pyrolysis of kraft lignin	244.5	0.2 A g^−1^	^48^
Carbon from pyrolysis of lignin	233	100 A g^−1^	^49^
Carbon from pyrolysis of bovine bone	134	10 mV s^−1^	^50^
Carbon from pyrolysis of Areca catechu husk	182	2 mV s^−1^	^51^
Carbon from pyrolysis of tea waste	167	4 A g^−1^	^52^
Carbon from pyrolysis of orange peel	168	0.7 A g^−1^	^53^
Carbon from pyrolysis of algae	241	5 A g^−1^	This work

**Figure 7:**
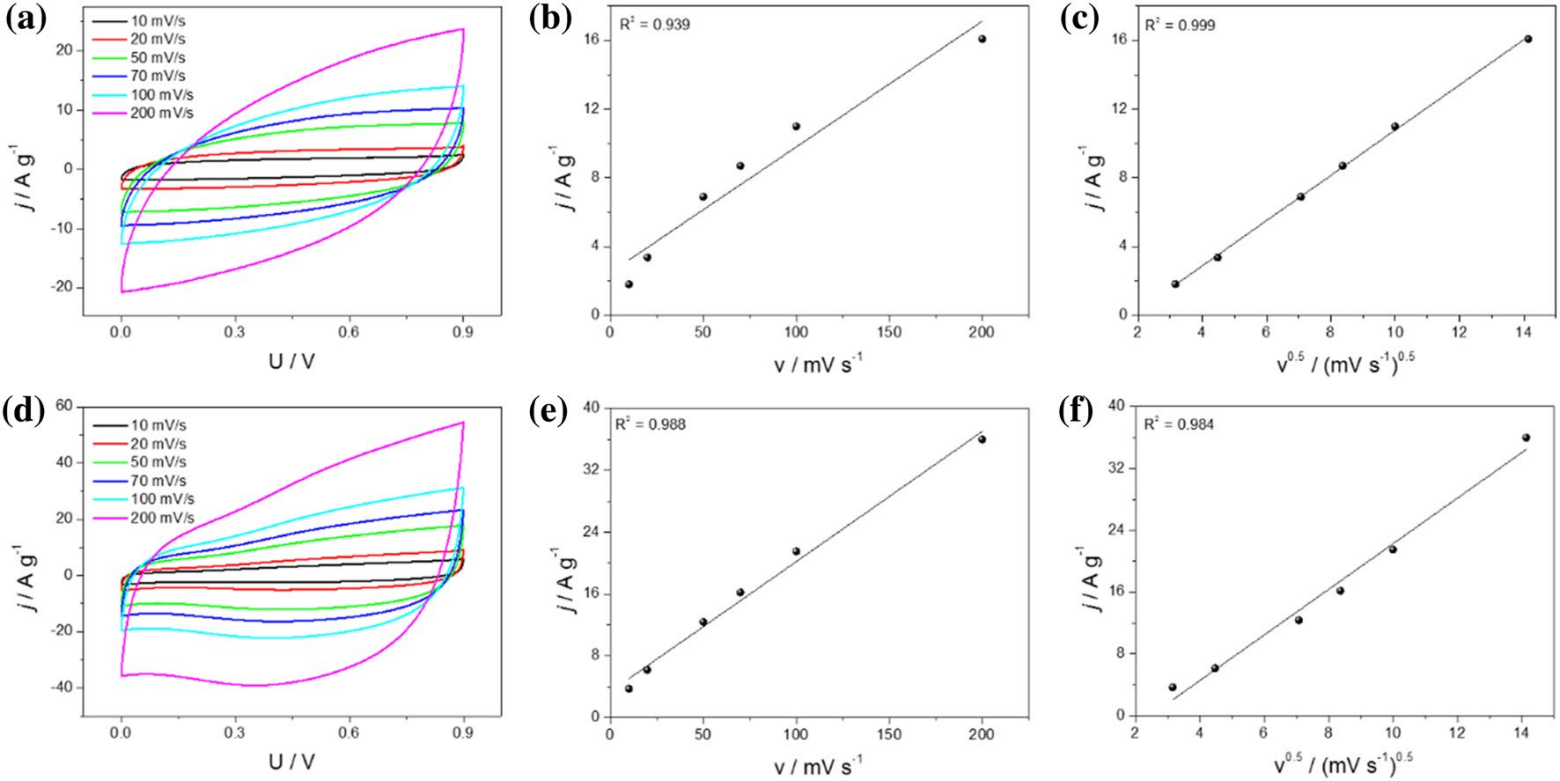
Cyclic voltammetry curves of (**a**) MLG and (**d**) AG electrodes in 0.2 M K_2_SO_4_ at different scan rates; (**b**,**e**) plots of *j* = f(*v*) and (**c**,**f**) j = f(v^1/2^) at *E* = 0.5 V for MLG and AG, respectively.

To delve into the underlying energy storage mechanism utilizing carbon-based electrodes, cyclic voltammetry was measured at varying scan rates. The obtained CV results are presented in Fig. 7a (for MLG) and Fig. 7d (for AG), encompassing a potential range from 0 to 0.9 V with scan rates of 10, 20, 50, 70, 100, and 200 mV s^−1^. To determine the charge transfer process mechanism, current density (j) versus potential (v) plots (Fig. 7b and Fig. 7e) were generated, including current density versus square root of the scan rate (v^1/2^) plots (Fig. 7c,f). These analyses were conducted at a specific potential value (E = 0.5 V) for both electrode materials.The fitting results for the MLG electrode material showed that the diffusion-controlled charge storage mechanism was the predominant contributor, which significantly outweighed the pseudocapacitance mechanism. In the case of AG, both mechanisms exhibited nearly equal responsibility for the charge accumulation process. This observation stems from the fact that charge storage occursat the electrode's surface and within the bulk material, suggesting a more complex and multifaceted energy storage mechanism.

**Figure 8:**
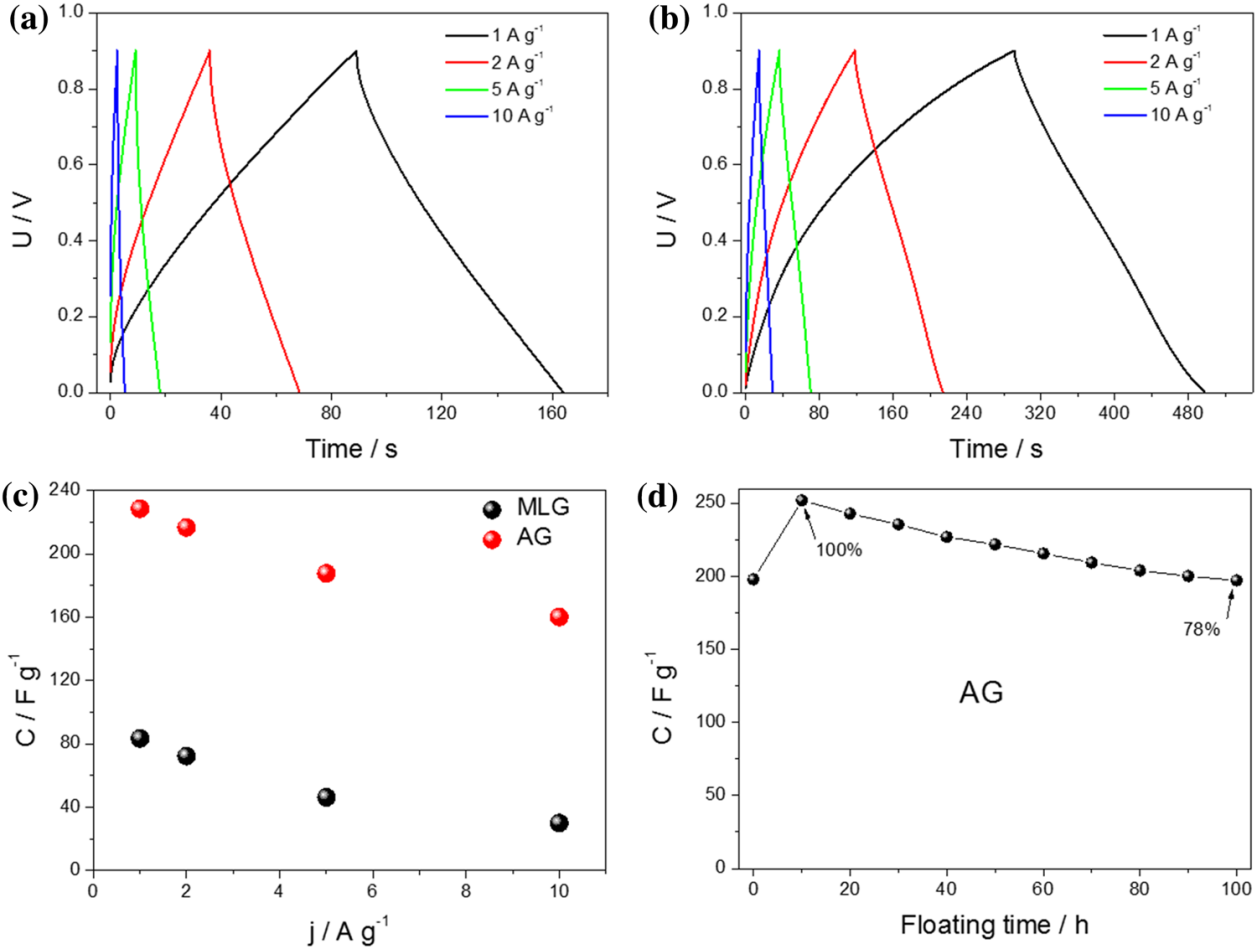
Charge and discharge curves at different current densities applied for (**a**) MLG symmetric supercapacitor and (**b**) AG symmetric supercapacitor. (**c**) The relationship between capacitance and charging/discharging current. (**d**) Effect of floating on the capacitance for the AG electrode.

Charge–discharge measurements were performed at various current densities for MLG and AG. Remarkably, both materials exhibited a symmetrical quasi-triangular shape even at high current densities, indicative of their high specific capacitance, as seen in Fig. 8a,b. This characteristic shape in the charge–discharge curves is highly desirable in supercapacitor applications as it signifies the ability to efficiently store and release energy. The quasi-triangular shape is associated with the rapid and reversible electrochemical processes occurring within the electrodes, allowing rapid charge and discharge cycles without significant energy loss. It is essential to note that varying charge–discharge times were observed in response to different current densities depending on the electrode material used. These distinct response times directly influenced the overall capacitance of the energy storage device. Furthermore, the accompanying graphs (Fig. 8c), which illustrate the relationship between capacitance and charge/discharge currents, reveal a similar trend of capacitance decrease with increasing current for both materials.

To ensure the accuracy and reliability of our stability assessment, the floating test was employed, which has emerged as a dependable technique for evaluating the long-term stability of electrochemical capacitors. This approach differs from conventional charge–discharge cycling over thousands of cycles. In this context, the symmetric cell was subjected to a floating test at 0.9 V and 5 A g^−1^ to assess the stability of the electrodes throughout their cycle life and to determine the point at which electrode degradation begins. The protocol involved a 10-h voltage holding period followed by five charge–discharge cycles, with this cycle repetition extending over 100 h. A preliminary test was performed to ensure the material's stability and readiness for the floating test. Prior to conducting the floating test, the material was subjected to a pre-conditioning process involving 100 cycles using cyclic voltammetry. This pre-conditioning step aimed to stabilize the material and prepare it for the subsequent floating test, which evaluated its long-term performance and durability. Figure 8d represents the regression of cell capacitance over the 100-h floating period. Despite the pre-conditioning process, it is worth noting that during the initial 10 h of the floating test, there was an observed increase in capacitance. The data from the graph demonstrate the stability of the cell voltage within this specified voltage window and duration, with a capacitance loss of approximately 22% (compared to the result after 10 h of floating). This observation underscores the enduring stability of the material even under prolonged operational conditions. It's important to note that this floating test was conducted specifically for the material that exhibited the highest capacitance, namely AG.

## Materials and methods

### Synthesis of carbon material from natural sources

Carbon materials were prepared using an uncomplicated pyrolysis method using natural sources (such as lysine and methylcellulose or the green algae – *Chlorella vurgalis*) and the cheap and widely available two hard templates K_2_CO_3_ and CaCO_3_. Carbon materials from natural sources were synthesized in two series. In the first series, natural carbon sources methylcellulose and lysine were used, along with a hard CaCO_3_ template in a weight ratio of 1:1:1. In the next step, a solution of K_2_CO_3_ at a concentration of 11% m/v was prepared and added to the mixture, which was then mixed mechanically and intensively until a homogeneous mass was obtained. In the second series, 100 mL of K_2_CO_3_ solution (concentration of 11% m/v) was added to 5 g of green algae and stirred until a homogeneous solution was obtained. In both series, the resulting mass was allowed to dry at 100 °C to evaporate water. The samples were then pyrolyzed at 800 °C for 1 h, with a heating rate of 10 °C min^−1^ in a nitrogen flow. After the pyrolysis process, the final product was treated with concentrated hydrochloric acid to remove residual carbonates. The samples were then washed with water and dried in an electric dryer. The second series was based on the use of the green algae, *Chlorella vurgalis*. The synthesis steps were analogous to the first series, and the mass ratio of the compounds was also the same. Commercial graphene nanoplatelets (G) with a specific surface area of 750 m^2^ g^−1^ and electrical conductivity of approximately 1,400 S cm^−1^ (purchased from Sigma Aldrich, Poland) were added to both series to improve the conductive properties at a mass ratio of 0.2 to the carbon material. The samples in the first series were marked as ML and MLG, where M was designed for methylcellulose and L for lysine used during the synthesis. The samples in the second series were named A and AG, where A is algae. In both series, G is commercial graphene.

### Solid state physics techniques

Surface analysis of the samples was conducted using a scanning electron microscope (SEM) type 1430 VP at an accelerating voltage of 30 kV from LEO Electron Microscopy Ltd, England. This SEM instrument allowed for detailed examination and characterization of the surface morphology and features of the materials. Raman spectra were recorded using a Raman spectrometer (Senterra, Bruker Optik) with a green laser (532 nm) as the excitation source. Raman spectroscopy allowed for the investigation of the materials' vibrational modes and molecular structure, providing insights into their chemical composition and bonding configurations. A detailed surface structure and features analysis was conducted to gain insights into the material's physical characteristics. The sorption of nitrogen was performed using an ASAP2020 Plus instrument from Micromeritics. Prior to the sorption measurements, the samples underwent a vacuum outgassing process at 200 °C for 24 h to remove any adsorbed contaminants. The specific surface area (S_BET_) was determined using the Brunauer–Emmett–Teller (BET) method, which calculates the surface area based on the adsorption isotherm data. Additionally, the total pore volume (V_total_) and pore size distribution were determined using the Density Functional Theory (DFT) method, providing information about the porosity of the carbon materials. To analyze the elemental composition of the samples, a bulk combustion analysis was conducted to measure the carbon, nitrogen, and hydrogen contents. This analysis provides quantitative information about the relative amounts of these elements present in the carbon materials. X-ray photoelectron spectroscopy (XPS) measurements were performed using a monochromatic Al Kα excitation source operated at 1486.6 eV. The survey spectra and high-resolution spectra were collected using pass energies of 0.5 eV and 0.1 eV, respectively. The spectra were referenced to the C1s neutral carbon peak at 284.8 eV, allowing for the characterization of the carbon-containing functional groups and their chemical states.

### Electrochemical measurements

The electrode materials were prepared by suspending 20 mg of the samples in a mixture of 0.75 mL distilled water, 0.2 mL isopropanol, and 0.05 mL of a 5% aqueous Nafion solution. The resulting suspension was sonicated in an ultrasonic bath for 60 min to ensure proper dispersion. Subsequently, the prepared mixture was deposited onto a polished surface of a glassy carbon electrode with a diameter of 1.5 mm and allowed to dry in an oven at 60 °C for a few minutes. For the electrochemical measurements, a three-electrode configuration was employed. The electrolyte used was a 0.2 M K_2_SO_4_ solution (κ ~ 0.3 S cm^−1^, pH ~ 7), with an Ag/AgCl (3 M KCl) reference electrode and a platinum mesh counter electrode. All electrochemical experiments were performed using a potentiostat/galvanostat (BioLogic VSP 2078) and involved cyclic voltammetry and galvanostatic charge–discharge (GCD) techniques. During the CV measurements, the potential was scanned from − 0.1 V to + 0.8 V vs. Ag/AgCl (3 M KCl) at a scan rate of 50 mV s^−1^. This range allowed for the investigation of the electrochemical behavior of the electrode materials. GCD tests were conducted within the same potential range, with a current density of 2.0 mA cm^−2^. The charge–discharge cycles provided insights into the energy storage capacity and performance of the synthesized materials. Moreover, EIS was performed for all obtained electrode materials in a frequency range between 20 kHz and 1 Hz with a voltage amplitude of 10 mV at an open circuit potential.

To construct a symmetric supercapacitor, two flexible graphite foils (GF) were combined with the obtained material electrodes. A fiberglass separator, soaked in a 0.2 mol L^−1^ K_2_SO_4_ aqueous electrolyte, was placed between the electrodes. The GF was coated with a mixture containing the tested carbon materials (MLG and AG), which was then dried at 40 °C for 6 h. The mass loading of the carbon materials onto the GF was determined by measuring the weight difference of the electrode material before and after applying the mixture. An analytical balance, specifically the RADWAG XA 82/220.4Y PLUS with an accuracy of 0.01 mg, was utilized for this measurement. The mass loading was found to be approximately 4.27 mg cm^−2^ for MLG and 5.38 mg cm^−2^ for AG. The thickness of the layers was about 30 µm. Subsequently, the casing foil was sealed around three sides using a plastic foil welder. Finally, the entire setup was securely sealed using a vacuum packing machine (CAS CVP-350/MS, Hertogenbosch). This construction process ensures the assembly of a symmetric supercapacitor with the MLG and AG materials as the electrodes. The use of flexible graphite foils provides mechanical support and flexibility to the device, whereas the fiberglass separator soaked in the electrolyte facilitates ion transportation between the electrodes while preventing direct contact. The sealing of the setup using the plastic foil welder and vacuum packing machine ensures the encapsulation and protection of the supercapacitor, maintaining its integrity and preventing moisture or air ingress. By employing these construction steps, a reliable and well-protected symmetric supercapacitor setup is achieved, allowing for further electrochemical characterization and performance evaluation of the MLG and AG materials. The galvanostatic charge and discharge tests (10,000 cycles) were performed for the symmetric supercapacitor. Charge and discharge measurements were made at a current density of 5 A g^−1^, with an electrochemical voltage range of 0 to 0.9 V. The specific capacitance (Csp) was calculated using the galvanostatic charge–discharge curves provided and Eq. (1):

1 $${C}_{sp}=\frac{I \cdot dt}{dV \cdot A (or m)}$$

where *I* is the applied discharge current, *t* is the discharge time, *V* is the discharge voltage and *A* is the active area in cm^2^ (m—mass of the active material).

The charge–discharge measurements were also carried out for MLG and AG at current densities ranging from 1.0 to 10 A g^−1^ over a polarization range of 0 to 0.9 V. A floating test was also conducted to evaluate the most promising stability for the carbon material, as detailed in reference^54^. The procedure involved conducting five galvanostatic charge–discharge cycles at maximum voltage after every 10 h of aging. The specific capacitance was then calculated. This series of sequences was repeated 10 times, totaling a floating time of 100 h.

## Conclusions

This article investigates the utilization of pyrolyzed natural compounds for supercapacitor applications, with a particular focus on the impact of graphene addition on the electrochemical performance. Four materials were studied: lysine with methylcellulose, lysine with methylcellulose-graphene composite, algae, and algae-graphene composite. The results demonstrate that incorporating graphene into the lysine-methylcellulose and algae matrices significantly improved the electrochemical properties of the resulting supercapacitor materials. Despite the graphene-infused materials having a smaller specific surface area compared to the carbon materials without graphene, they exhibited enhanced capacitance. The superior electrochemical performance observed in the graphene-enhanced materials can be attributed to several factors. Firstly, graphene, with its high electrical conductivity, facilitates efficient charge transfer and electron transport within the electrode. This results in improved capacitive behavior and enhanced energy storage capacity. Furthermore, the graphene reinforcement provides mechanical stability to the electrode materials, preventing structural degradation during repeated charge–discharge cycles. The resulting electrodes exhibit excellent cyclic stability and retain their electrochemical performance over extended operation. Thus, the findings of this study highlight the significant role of graphene in enhancing the electrochemical properties of pyrolyzed natural compound-based supercapacitor materials. Despite having a smaller specific surface area, the addition of graphene leads to improved capacitive behavior, higher specific capacitance, and excellent cyclic stability.

## Data Availability

The datasets generated and/or analyzed during the current study are available in the BRIDGE OF KNOWLEDGE repository (https://mostwiedzy.pl/pl/open-research-data/raman-spectra-for-pyrolized-natural-compounds, 80807500181120-0).
